# Mapping Surface‐Defect and Ions Migration in Mixed‐Cation Perovskite Crystals

**DOI:** 10.1002/advs.202404468

**Published:** 2024-08-29

**Authors:** Razan O. Nughays, Khulud Almasabi, Sarvarkhodzha Nematulloev, Lijie Wang, Tieyuan Bian, Issatay Nadinov, Bahaaeddin Irziqat, George T Harrison, Shadi Fatayer, Jun Yin, Osman M. Bakr, Omar F. Mohammed

**Affiliations:** ^1^ Advanced Membranes and Porous Materials Center (AMPM) Division of Physical Science and Engineering King Abdullah University of Science and Technology Thuwal 23955‐6900 Kingdom of Saudi Arabia; ^2^ KAUST Catalysis Center Division of Physical Sciences and Engineering King Abdullah University of Science and Technology Thuwal 23955‐6900 Kingdom of Saudi Arabia; ^3^ Functional Nanomaterials Lab Division of Physical Sciences and Engineering King Abdullah University of Science and Technology Thuwal 23955‐6900 Kingdom of Saudi Arabia; ^4^ Department of Applied Physics The Hong Kong Polytechnic University Kowloon Hong Kong 999077 P. R. China; ^5^ KAUST Solar Center (KSC) Division of Physical Science and Engineering King Abdullah University of Science and Technology Thuwal 23955‐6900 Kingdom of Saudi Arabia

**Keywords:** DFT, electron imaging, ion migrations, perovskites, surface

## Abstract

Single crystal perovskites have garnered significant attention as potential replacements for existing absorber layer materials. Despite the extensive investigations on their photoinduced charge‐carriers dynamics, most of the time‐resolved techniques focus on bulk properties, neglecting surface characteristic which plays a crucial role for their optoelectronic performance. Herein, 4D ultrafast scanning electron microscopy (4D‐USEM) is utilized to probing the photogenerated carrier transport at the first few nanometers, alongside density functional theory (DFT) to track both defect centers and ions migration. Two compositions of mixed cation are investigated: FA_0.6_MA_0.4_PbI_3_ and FA_0.4_MA_0.6_PbI_3_, interestingly, the former displays a longer lifetime compared to the latter due the presence of a higher surface‐defect centers. DFT calculations fully support that revealing samples with higher FA content have a lower energy barrier for iodide ions to migrate from the bulk to top layer, assisting in passivating surface vacancies, and a higher energy diffusion barrier to escape from surface to vacuum, resulting in fewer vacancies and longer‐lived hole–electron pairs. These findings manifest the influence of cation selection on charge carrier transport and formation of defects, and emphasize the importance of understanding ion migrations role in controlling surface vacancies to assist engineering high‐performance optoelectronic devices based on single crystal perovskites.

## Graphical abstract

Comprehensive investigation of the surface of mixed‐cation perovskites demonstrated using 4D‐ultrafast scanning electron microscopy (USEM) and density functional theory (DFT) calculations. From the former, higher formamidinium (FA) content lead to long‐lived carrier lifetime compared to methylammonium (MA). DFT reveals that iodide ion migration from bulk to surface in FA‐rich assists in eliminating surface vacancies and enhance surface quality.

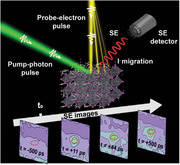

## 1 Introduction

The electronic and optical properties of absorber layers are critical factors that determine the efficiency of solar cell devices. Polycrystalline perovskites exhibited outstanding optoelectronic properties, ranging from long charge carrier lifetimes to feasible bandgap tunability.^[^ ^1^, ^2^ ^]^ They have demonstrated an impressive power conversion efficiency (PCE) of 26.7%.^[^ ^3^ ^]^ These features enable polycrystalline perovskites to compete with well‐established semiconductors such as gallium arsenide (GaAs) and silicon (Si).^[^ ^4^, ^5^, ^6^ ^]^ However, further improvements have been challenging due to the grain boundaries that host a high concentration of defect states that could significantly affect the overall device performance.^[^ ^7^, ^8^ ^]^ Deviation towards grain‐free single‐crystal perovskites, particularly single‐crystal MAPbI_3_ and FAPbI_3_, is proven to be a promising direction to overcome this obstacle.^[^ ^9^ ^]^ The general formula of hybrid perovskites is ABX_3_, where A represents a cation, B is a metal ion and X stands for halides, typically, formamidinium (FA) or methylammonium (MA) are the most common selection for organic cations.^[^ ^10^ ^]^ For instance, a long charge carrier diffusion length of 175 micrometers (µm) in the bulk of single crystal MAPbI_3_ and 22 µm on the surface have been reported in comparison to its polycrystalline counterparts with a diffusion length of 1 µm.^[^ ^11^, ^12^, ^13^ ^]^ Moreover, single crystal FAPbI_3_ has achieved a bandgap close to the optimal Shockley‐Queisser limit of 1.48 eV.^[^ ^6^, ^14^ ^]^ Despite all mentioned advancements, a few challenges have emerged that necessitate attention: 1) the volatility of the MA cation makes it thermally unstable,^[^ ^15^ ^]^ 2) the sudden phase transition in FAPbI_3_ from the perovskite phase (α‐FAPbI_3_) to the non‐perovskite δ‐FAPbI_3_ at room temperature remains ambiguous,^[^ ^16^, ^17^ ^]^ and, more importantly, 3) the elevated density of surface defect and ion migration that result in distortion in the electronic band structure, directly impacting charge carrier dynamics.^[^ ^18^, ^19^ ^]^

In this context, another approach involving alloying the cation site in single crystals led to numerous successful attempts in overcoming some of the aforementioned problems.^[^ ^20^, ^21^ ^]^ It has been recognized that the variation in properties are strongly influenced by the dominant cation and cation's size, which also control the stability of the crystals and alter its electronic structure. By substituting some of the large cation (FA^+^) in FAPbI_3_ with the smaller MA^+^, the perovskites phase stabilizes significantly.^[^ ^22^ ^]^ Previous reports showed that the tolerance factor almost reached 1 and the lifetime notably enhanced when increasing FA content from 0 to 0.6.^[^ ^23^, ^24^ ^]^ The mixed‐cation single crystals with higher FA contents have demonstrated superior performance for solar cells achieving a PCE of 23.1%,^[^ ^25^ ^]^ setting a new world record for single crystal perovskite solar cells. However, surface defect and ion migration remain problematic. To address this limitation, significant efforts have been dedicated to surface treatment, engineering and passivation to alleviate these deformities.^[^ ^9^, ^26^, ^27^ ^]^ Therefore, a comprehensive investigation of the surface and interface is essential to provide a profound understanding of the influence of cation selection on charge carrier behavior at the uppermost layers and the contribution of ion migration in this regard.^[^ ^28^ ^]^

Transient absorption spectroscopy and microscopy (TAS and TAM) are powerful pump‐probe techniques that provide valuable dynamical insights into the charge carrier kinetics and diffusion lengths.^[^ ^29^, ^30^ ^]^ Where TAS can provide a deeper understanding of fundamental phenomena, such as the contribution of phonon into photo‐induced bandgap normalization in perovskites, it lacks the spatial components that can be accessed using TAM.^[^ ^31^, ^32^ ^]^ However, these techniques are limited by the relatively high penetration depth of the probe beam which in turn offers information primarily from the bulk material (micrometers to tens of nanometers), neglecting the surface information.^[^ ^33^ ^]^ There are other surface‐sensitive techniques like X‐ray photoelectron spectroscopy (XPS) and scanning probe microscopy (SPM) that able to image in atomic level spatial resolution.^[^ ^34^, ^35^ ^]^ However, one of their major drawback is the lack of capturing charge carriers events in real time with high temporal resolution. Only the state‐of‐art 4D ultrafast scanning electron microscopy (4D‐USEM) excels in selectively investigating the photophysical processes on the surface of materials with excellent spatiotemporal resolutions at the nanometer (nm) and femtosecond (fs) scales, respectively.^[^ ^36^, ^37^ ^]^ In this technique, a photon‐pulse of 515 nm is employed to pump the sample, and further synchronized with accelerated beam of packets of pulsed‐electrons, inside the microscope, that used to probe the specimen's surface by collecting secondary electron (SE). The photon‐pump pulse leads to a change in the contrast in the illuminated area, allowing for tracking photo‐induces charge carrier in real space and real time simultaneously. It should be noted that 4D‐USEM has been actively utilized to investigate charge carrier dynamics at the first few nanometers (≈5 nm), understand the fundamental cause of signal‐contrast, and probe diffusion lengths at the surface for different types of semiconductors.^[^ ^12^, ^31^, ^38^, ^39^ ^]^

In this study, we explore and decipher the influence of cations selection on surface‐defects, ion migration and charge carrier behavior of mixed‐cation single‐crystal perovskites. Our approach involved the utilization of 4D‐USEM on two compositions: FA_0.6_MA_0.4_PbI_3_ and FA_0.4_MA_0.6_PbI_3_ to probe surface‐defects, as well as tracking ion migration using advanced density function theory (DFT) calculations. Both compositions exhibited a dark signal contrast in 4D‐USEM images, and a fast formation of charge carrier after photoexcitation. Notably, FA_40_MA_60_PbI_3_ demonstrated a faster recovery of charge carriers within 1 ns compared to FA_60_MA_40_PbI_3_. DFT calculations revealed that the iodide ions in pristine MAPbI_3_ have a lower migration energy barrier to escape the outermost layer of leaving vacancies, suggesting higher defect densities in the former case. On the other hand, iodide ions in pristine FAPbI_3_ and mixed‐cation have a lower energy barrier from the bulk to surface, and a higher energy diffusion barrier from surface to vacuum. In other words, the iodide ions act to passivate surface‐vacancies as they diffuse from the subsurface to the top‐surface, resulting in fewer vacancies and longer‐lived hole–electron pairs. Our findings provide a comprehensive investigation, spanning from experimental to theoretical approaches, on cations’ selection role in charge carriers and its contribution in ion migration. Motivating efforts to understanding charge carrier transport and expand surface treatment approaches to overcome current challenges.

## 2 Results and Discussion

The mixed‐cation single crystal perovskites were prepared using the inverse temperature crystallization method (ITC), as previously reported.^[^ ^22^ ^]^ Two compositions of mixed‐cations were synthesized: FA_0.6_MA_0.4_PbI_3_ and FA_0.4_MA_0.6_PbI_3_. For simplicity: “FA‐rich” is used to refer to the former, and “MA‐rich” is used to the latter. For the preparation method see Experimental section in Section 4.

### 2.1 Structure Characterization of the Two Compositions

**Figure 1:**
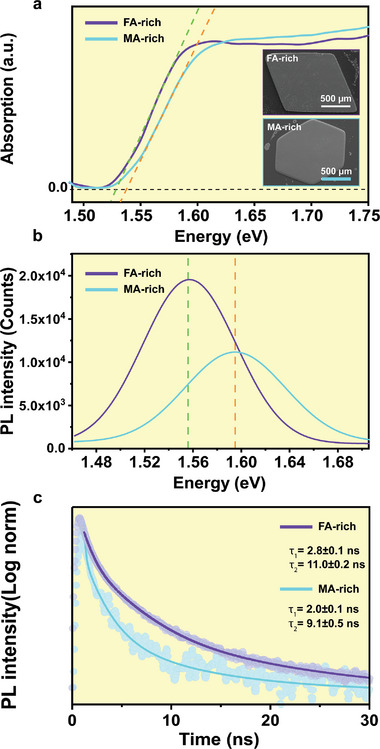
a) Absorption coefficient of the two compositions obtained by VASE at room temperature. The dashed lines represent an estimation of the optical bandgap position of each sample (green for FA‐rich and orange for MA‐rich), which are in between the values of pure‐FAPbI_3_ and pure‐MAPbI_3_ showing the redshift of the bandgap. The inset show static SEM images for the two mixed cation crystals. FA‐rich in the top with a quadrilateral shape and MA‐rich (bottom) with hexagonal shape. b) PL peaks for FA‐rich with higher PL intensity (in purple) and MA‐rich (cyan) demonstrating a lower PL due the presence of higher defect centers. The dashed lines in orange (MA‐rich) and green (FA‐rich) also indicate of the red‐shift with varying the composition. c) Demonstrates the TRPL for FA‐rich and MA‐rich with two time components. MA‐rich exhibited relatively a shorter lifetime compared to FA‐rich.

To correlate the changes in optical properties with crystal compositions, **Figure** **1**a shows steady‐state absorption spectra of the two samples obtained by variable‐angle spectroscopic ellipsometry (VASE). Note that two focusing beams were used at different angles of 60°, 65°, and 70° to ensure accuracy. The spectra depict typical absorption spectra for FA‐rich (in purple) and MA‐rich (in cyan). The dashed lines in green and orange indicate the estimated optical bandgap for FA‐ and MA‐rich, and the inset shows static SEM images for the two compositions. As observed in the figure, the absorption spectra positioned between pure‐MAPbI_3_ (1.55 eV) and pure‐FAPbI_3_ (1.48 eV), confirming the tunability of the bandgap by varying the cation size and composition, where FA‐rich tends to have wider absorption in near IR spectral range.

The inset in Figure 1a reveals SEM images of the morphology and surface quality for FA‐rich (top) and MA‐rich (bottom) with a scale bar of 500 µm. The two compositions exhibit distinct structures, with FA‐rich forming a quadrilateral shape, and MA‐rich adopting a hexagonal shape (see Figure S1, Supporting Information). It is worth mentioning the difficulty of maintaining a good surface quality for single crystals perovskite. Figure S2a (Supporting Information) illustrates different crystals with poor surface‐quality, displaying solvent residues, particles that originated during synthesis and crystals degradation when exposed to air for some time. These crystals and areas were intentionally avoided during the measurements. Figure S2b (Supporting Information) provides examples of the regions selected for image acquisitions.

Steady‐state photoluminescence (PL) was performed upon laser excitation at 405 nm at room temperature. Each composition displays a single PL peak with a small red‐shift in the center emission for FA‐rich which is consistent with the absorption spectra (Figure 1a). The PL peaks are located at (1.56 eV)—and (1.59 eV)—for FA‐rich and MA‐rich, respectively (Figure 1b). The red‐shift observed in FA‐rich has been reported and discussed previously in details with increasing FA contents, and it has been attributed to the narrower bandgap observed above due to modification of the electronic structure.^[^ ^23^ ^]^ Moreover, a lower PL intensity for MA‐rich is observed, implying of higher number of defect states in this composition. Table S1 (Supporting Information) shows the energy formation for vacancies on pure‐FA and pure‐MA calculated by DFT under different conditions (I‐rich, moderate and Pb‐rich). From the table, in all scenarios, MA has a lower formation energy for vacancy, directly leading to more surface defects than FA.

Figure 1c illustrates the time‐resolved PL (TRPL) decay curves for FA‐rich and MA‐rich fitted with a bi‐exponential decay function. It is worth noting that the signal of TRPL is a mixture of contributions from the surface and the bulk regions in the sample, and it can be differentiated by their characteristic decay times. Two time decay components are observed in the TRPL kinetics, a fast decay (*τ*_1_) component with fitted values of 2.8 ± 0.1 and 2.0 ± 0.1 ns, and a slow decay component (*τ*_2_) has corresponding values of 11.0 ± 0.2 and 9.1 ± 0.5 ns for FA‐rich and MA‐rich, respectively. The fast component is assigned to carriers lifetime at the surface, while the longer‐lived component represents the lifetime of carriers propagating into the bulk.^[^ ^18^, ^40^ ^]^ Note that the fitted average lifetime (τ_avg_) values for FA‐rich and MA‐rich samples are 4.3 and 3.6 ns, respectively. Figure S3 (Supporting Information) presents the time decay of three other areas of the two compositions.

### 2.2 Surface Defects and Carrier Dynamics

**Figure 2:**
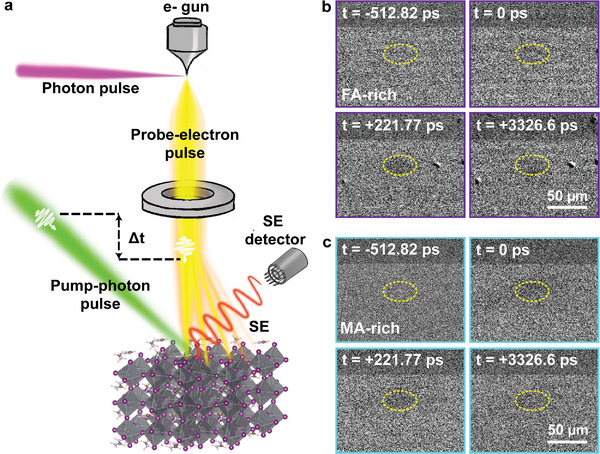
a) Simplified illustration of 4U‐SEM technique, where the pump‐pulse is guided toward the sample to initiate the dynamics and the UV beam is directed to the tip of the electron gun source inside the microscope to generate beam of pulsed electron utilized for probing pixel by pixel. A time difference is introduced between the two pulses by controlling the arrival of the pump‐pulse through a mechanical optical delay line. Time‐resolved SE snapshots of b) FA‐rich and c) MA‐rich exhibiting a dark image contrast after photo‐excitation in both samples. The yellow ellipse indicates the excitation beam‐size (40 µm).

**Figure** **2**a illustrates the concept of the pump‐probe 4D‐USEM that integrates SEM with a femtosecond fiber laser system. More details about the set‐up can be found in the Experimental section and elsewhere.^[^ ^37^ ^]^ Briefly, 515 nm (green) photon pump‐pulse is directed to the sample inside SEM chamber to optically excite it and initiate dynamics, and further synchronized with pulsed‐electron beam, generated from directing a 343 nm (ultraviolet, UV) to the gun system inside the microscope, to probe the sample instead of the continuous thermal electrons in the conventional SEM experiments. Upon the interaction of the pulsed primary electrons with the specimen, secondary electrons (SE) are produced from the uppermost layer of the sample, and collected by SE detector “Everhart‐Thornley.”^[^ ^41^ ^]^ The time difference between the pump‐pulse and probe‐pulse is controlled mechanically by optical delay line that introduce additional distance to the pump path. Generally, the images obtained exhibit one of the two contrasts on the illuminated area (by the pump): a) a bright contrast in case of energy gain mechanism; portraying a higher probability of emitting SE, or b) dark contrast when SEs lose energy due to multiple reasons, that will be discussed below, resulting in a decrease of SE emission.^[^ ^36^, ^37^ ^]^ We performed 4D‐USEM on the two crystals of two different compositions, Figure 2b,c displays time‐resolved images of SE for FA‐rich and MA‐rich, respectively. The yellow ellipse in the center represents the size and position of the pump beam on the crystal surface, the negative sign refers to the time when the probe‐pulse beam arrives to the sample prior of the pump‐pulse beam, and no contrast change is observed in this region. While the positive sign refers to the time when the pump‐pulse beam reaches the sample first and initiates the dynamics, resulting in an image contrast change. Both compositions exhibited a dark contrast after photo‐excitation (see also Figure S4, Supporting Information). It should be noted that the dark contrast forms when SE lose some energy as they escape the surface, various phenomena are accountable for that, such as: a) work function increases after photoexcitation due to the presence of oxidation layers, b) band bending that leads to accumulation of charged electrons that reduce SE collection efficiency and c) different collisions events that hinder SE emission as they migrate out of the surface.^[^ ^12^, ^42^, ^43^, ^44^, ^45^ ^]^ In our case, it conveys the presence of surface defects, such as vacancies, results in charge imbalance and structural alteration.^[^ ^46^, ^47^ ^]^ To confirm this, X‐ray photoelectron spectroscopy (XPS) have been performed on as‐received samples, and after 8 h of light exposure. Following light exposure, we observed the formation of an additional peak around 136.7 eV, indicating the generation of metallic lead (Pb^0^). Figure S5 (Supporting Information) demonstrates the reduction of lead ions (Pb^2+^) to Pb^0^, which suggests a loss of iodide ions in the form of methylammonium iodide (MAI).^[^ ^48^ ^]^ Furthermore, we noted a slight peak shift in FA‐rich and a significant shift in MA‐rich sample, serving as a direct evidence of iodide ion migration altering the structure. Table S2 (Supporting Information) presents XPS results under light exposure in a nitrogen environment, revealing a higher iodide loss in MA‐rich sample compared to FA‐rich sample. Additionally, we observed significant reduction in the Pb/I ratio, confirming a greater loss of iodide ions (I^−^). More details about ions pathways are discussed in later section. Probing poor surfaces can lead to different image contrast of SE, hence misinterpretation of the mechanism. For instance, Figure S6 (Supporting Information) presents a bright contrast for FA‐rich which mean energy‐gain mechanism, however by inspecting the probed area there is some solvent residue and surface degradation of the sample. While MA‐rich presented the two contrast, weak dark image‐contrast or bright contrast with slow formation as can be seen in Figure S7a,b (Supporting Information), respectively.

**Figure 3:**
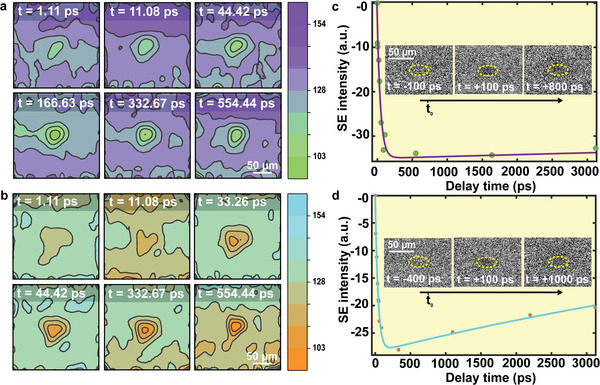
a,b) Color‐coded 4D‐USEM images at different delay time after photoexcitation for FA‐rich and MA‐rich, respectively. The green and orange color in the center refer to the illumnated region by the pump‐beam that resulted in different. The scale bar is 50 µm. c,d) Display the charge‐carrier dynaimcs of FA‐rich and MA‐rich. Both compositions exhibited a fast formation of charge carrier within ≈100 ps then it starts to recover (after ≈200 ps). However, MA‐rich showed a faster recovery reflecting a faster recombination of the photo‐induced carriers. The negative y‐axis stands for the dark image contrast.

To further investigate the influence of the dominant cation (FA or MA) on surface defects and its impact on charge carriers, a color‐coded 4D‐USEM images are demonstrated in **Figure** **3** at different time delays: ≈1, ≈11, ≈44, ≈166, ≈330 and ≈554 ps. The images were fitted using the 2D Gaussian function to enhance signal‐to‐noise ratio. The green and orange colors at the center of Figure 3a,b represent SE signal for FA‐rich and MA‐rich, respectively. As we can see in the figure, the signal starts to form immediately after photo‐excitation, however, an obvious and fast recovery was observed after some time, especially for MA‐rich. In Figure 3c,d, we plotted the difference in SE intensity as a function of the delay time between the pump‐photon beam and the electron‐probe beam (up to 3 ns) to examine the dynamics of the charge carriers. The data were extracted from a square area in the original 4D‐USEM images (see Figure S8, Supporting Information), and then fitted with two exponential functions to show the dynamical trend of the two compositions. After laser excitation, both crystals have exhibited a sharp drop in SE intensity at the illuminated area, ascribed to an energy loss mechanism as mentioned before. After ≈200 ps, the curve is almost flattened in FA‐rich (Figure 3c) reflecting a slow recovery. On the other hand, a fast decay was observed in MA rich with a similar time scale of 200 ps (Figure 3d), affirming the high number of defect states that accelerate the charge carrier recombination processes, showing consistency with the TRPL results discussed earlier.

### 2.3 Ions Migration Pathways

**Figure 4:**
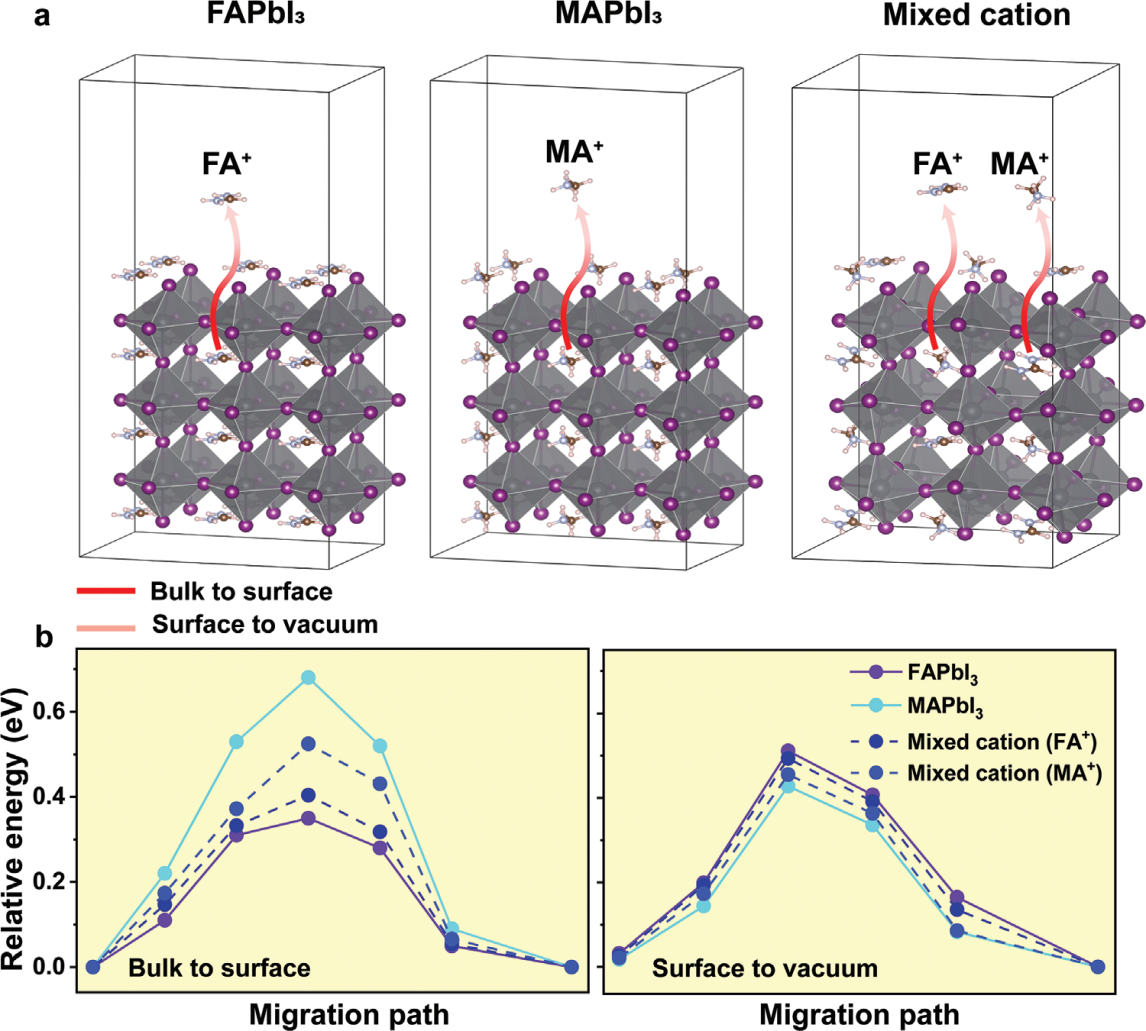
a) Side‐view of migration pathway for the cations ion (FA^+^ and MA**^+^**) in pristine FAPbI_3_, pristine MAPbI_3_ and mixed cation of 50/50 ratio from bulk to surface (in red) and surface to vacuum (in pink) as indicated by the arrows. b) Energy barrier for cations ions diffusion in two different regimes, bulk to surface and surface to vacuum, for pure‐FAPbI_3_, pure‐MAPbI_3_ (purple and cyan) and the mixed cation (in blue dashed lines).

**Figure 5:**
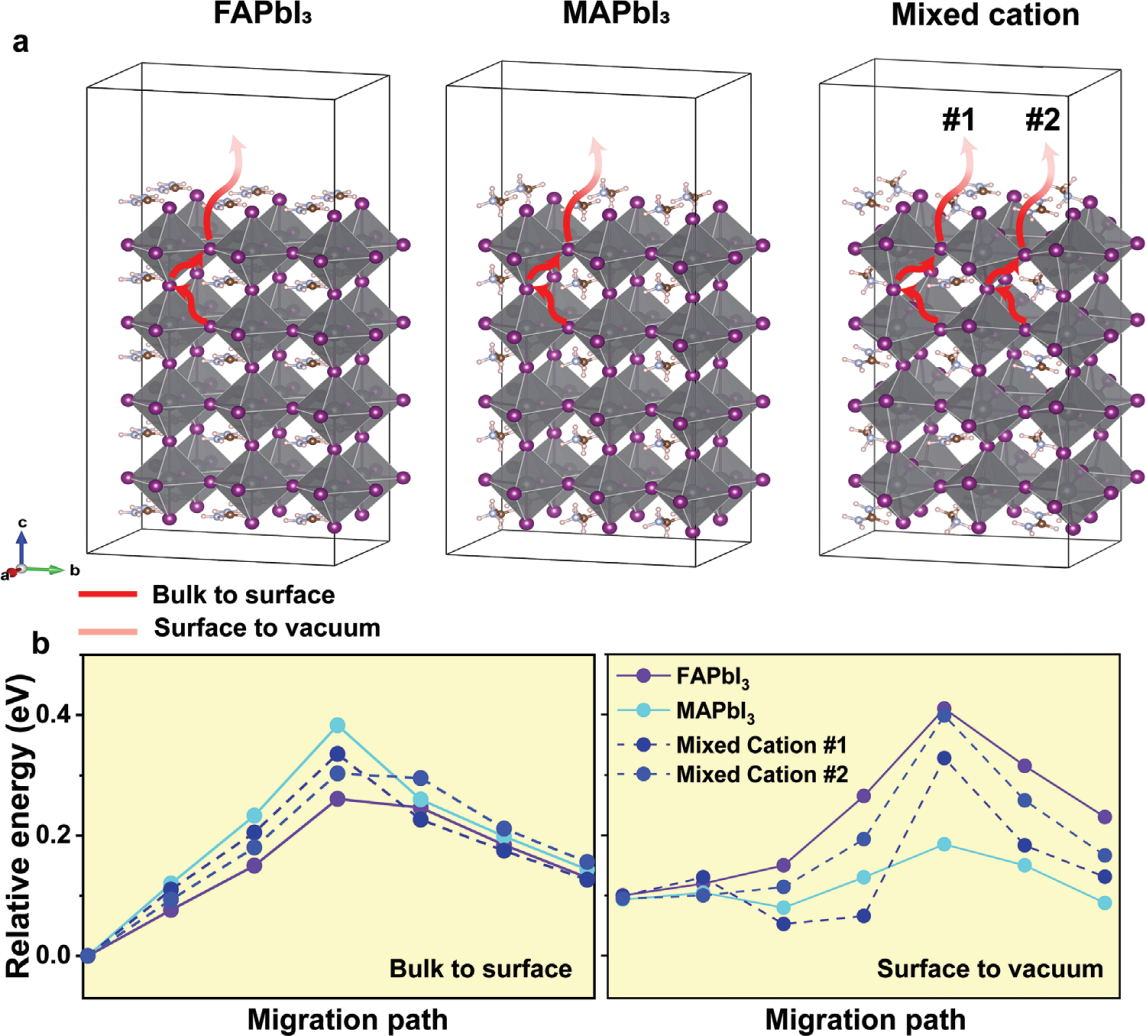
a) Side‐view of migration path for I**^−^** ion in the three different compositions. There are two paths in mixed cation for the migration through FA^+^ (#1) and through MA^+^ in path #2. b) Energy barrier of iodide ions from bulk to vacuum revealing that I^−^ ion in FA‐rich composition demonstrates the lowest energy from bulk to surface, and the highest energy from surface to vacuum disallowing it from escaping the surface and forming vacancy.

To investigate the experimental findings with the impact of cations’ selection on ions migration, we carried out DFT calculations to track ion migration from the bulk of material to surface, and from surface to vacuum on pristine FAPbI_3_, representing FA‐rich condition, pristine MAPbI_3_ for MA‐rich and on mixed cation with 50/50 ratio (FA_0.5_MA_0.5_PbI_3_) in order to simplify the analysis (refer to Figure S9, Supporting Information for the unit cell of mixed cation). We investigated the migration pathways for FA^+^ and MA^+^ ions, along with iodide (I^−^), identified as the most mobile ions in halide perovskites.^[^ ^49^ ^]^ **Figure** **4** showcases the diffusion energy barrier of the cations ions (FA^+^ and MA^+^) in pristine compositions and the mixed cation from the bulk to surface and surface to its surrounding, respectively. Interestingly, FA^+^ ions demonstrated the lowest energy from bulk to surface, while MA^+^ had the highest energy. As the ions travel from surface to the top‐surface, the diffusion energies change from lowest to highest in case of FA^+^ cations, and the opposite for MA^+^, implying that MA ions leave some vacancies on the surface (see Figure S10, Supporting Information, for another slap). Similarly, iodide ions migrations demonstrated a significant change in the energy depending on the composition and the region. **Figure** **5**a reveals I^−^ ions diffusion pathways from the bulk until escaping the surface, as indicated by the red/pink arrows, in FAPbI_3_, MAPbI_3_ and mixed cation. For the mixed cation, the I^−^ ion has two paths depending on the neighboring cation illustrated by (#1) for FAPbI_3_ and (#2) for MAPbI_3_. The energy barrier for iodide is depicted in Figure 5b, determined by the crystals composition and the regime, the energy barrier can vary significantly. For instance, I^−^ in FAPbI_3_ exhibited the lowest energy as it diffused from the bulk to the surface with a value of 0.26 eV, however, it increased to 0.41 eV as it moved from the surface to its surrender. Contrary, iodide ions in MAPbI_3_ presented a high energy barrier in the bulk (0.383 eV) and the lowest energy on the surface (0.185 eV) that could result in formation of surface vacancies (see Figure S11, Supporting Information).

When considering the mixed cation case of 50/50 ratio, the energy barrier for I^−^ ion displays a relatively close energy values from bulk to surface and surface to vacuum, suggesting that the behavior of I^−^ ion depends on the dominant cation. In other words, if the composition contains more FA the iodide ion tends to behave similar to the I^−^ in pristine FAPbI_3_ scenario, and more toward MAPbI_3_ if it has higher MA ratio than FA. Moreover, it reveals more facile I^−^ ion migration for crystals with higher FA^+^ contents, assisting surface‐passivation by the ions as they diffuse from the bulk to the surface that could eliminate surface vacancies, and then encountering a higher energy preventing it from escaping the surface. Figure S12 (Supporting Information) displays I^−^ ion migration from bulk to surface for another slap, indeed iodide ion from the bulk to the surface in FAPbI_3_ showed a lower energy diffusion barrier of 0.12 eV. Previous research highlighted the effectiveness of I^−^ passivation when paired with suitable cations that form strong interactions, resulting in an increased energy barrier for iodide ion.^[^ ^50^ ^]^ These results provide insights into the role of the cation unit on the ion migration in perovskites single crystals.

## 3 Conclusion

In summary, we investigated the photo‐induced charge‐carrier transport properties of two compositions of mixed‐cation single crystals perovskite, FA‐rich and MA‐rich, using 4D‐USEM and advanced DFT calculations. We demonstrated that FA‐rich exhibited a long‐lived charge carriers, while MA‐rich experienced a faster charge carrier recombination. This could be attributed to the presence of higher surface defects that hinder the charge‐carrier transport in MA‐rich compared to crystals with higher FA content. Being in this regime, DFT evidenced that FA‐rich crystals not only have a lower energy for ion migrations from the bulk to the top‐layers, thereby facilitating surface‐passivation by eliminating, to some extent, vacancies site, but also a higher energy barrier to migrate from the surface to its surroundings. These findings underline the significance of the cation selection on the charge carrier dynamics and ions migration at the first few nanometers of the top surface and uncover their contribution on the whole performance of the device.

## 4 Experimental Section

### Sample Fabrication: Materials

Methylammonium iodide (MAI) and formamidinium iodide (FAI) were taken from Greatcell Solar Limited (Australia). Lead(II) iodide (PbI_2_, ultradry, 99.999%) was taken from Alfa Aesar. γ‐Butyrolactone (GBL, >99%) was taken from Sigma Aldrich. Poly(triarylamine) (PTAA) was taken from Xi'an Polymer Light Technology. All materials were used as received without further purification. Conductive indium tin oxide (ITO) glass was taken from Delta Technologies LTD, CO, USA.

### Methods

The cleaning starts with sonication of ITO glass substrates measuring 5 cm × 5 cm in detergent, DI water, acetone, and isopropyl alcohol sequentially for 10 min each, followed by surface treatment of ultraviolet‐ozone (UVO) for 15 min. Next, 400 µL of PTAA solution (2.5 mg mL^−1^ in toluene) was spin‐coated for 30 s at 4000 rpm and subsequently annealed for 10 min at 100 °C.

### FA_0.6_MA_0.4_PbI_3_ Single Crystal Growth

An equimolar amount of FAI and MAI (0.6 FAI + 0.4 MAI) and PbI_2_,1.9 m solution in GBL was dissolved by stirring overnight at 50 °C to prepare the FA_0.6_MA_0.4_PbI_3_ precursor_._ Approximately 60 µL of the perovskite solution was placed on a PTAA‐coated substrate at 50 °C and sandwiched with another PTAA‐coated substrate. The temperature was then raised to 75 °C at a rate of 15 °C h^−1^ and then to 130 °C at a rate of 3 °C h^−1^ to induce nucleation and growth.

### FA_0.4_MA_0.6_PbI_3_ Single Crystal Growth

An equimolar amount of FAI and MAI (0.4 FAI + 0.6 MAI) and PbI_2_ 1.6 m solution in GBL was dissolved by stirring overnight at 60 °C to prepare the FA_0.4_MA_0.6_PbI_3._ Approximately 60 µL of the perovskite solution was placed on a PTAA‐coated substrate at 60 °C and sandwiched by another PTAA‐coated substrate. The temperature was then raised to 80 at a rate of 20 °C h^‐1^ and then to 130 °C at a rate of 3 °C h^‐1^ to induce nucleation and growth.

After the crystallization was terminated, the substrates were cooled and separated using a razor blade, and the remaining solution was cleaned with Kimwipes. The substrates were left to cool slowly to room temperature on the hotplate.

### Variable‐Angle Spectroscopic Ellipsometry (VASE)

Spectroscopic ellipsometry was performed using an M‐2000 DI device (J. A. Woollam, USA), which operated in the 193–1690 nm wavelength range. The sample was measured at a minimum of three angles of incidence (65°, 70°, and 75°), and the data analysis was performed using the Complete EASE 6.51 software package, to generate the absorption coefficient of perovskite signal crystal.

### Time‐Resolved Photoluminescence (TRPL)

For the time‐resolved photoluminescence (PL) experiments, the FA_60_ MA_40_ PbI_3_ and FA_40_ MA_60_ PbI_3_ single crystals on ITO glass were excited using a pulsed diode laser at 405 nm. This laser, with a pulse duration of 90 ps, was provided by a HORIBA Delta Diode and was precisely focused through a 20×, 0.38 numerical aperture (NA) objective on a customized Olympus IX71 microscope. To ensure complete relaxation between excitation pulses, the interpulse duration was set to exceed the PL decay time (10 MHz). The pulse intensity was finely controlled by utilizing a series of neutral density filters from Thorlabs to guarantee that less than 1% of the excitation events resulted in the detection of a single photon. A long‐pass 450 nm filter from Newport was employed to reject scattered laser light and selectively choose the emission wavelength for monitoring. The filtered PL signal was directed and focused onto an avalanche photodiode (PDM series, MicroPhoton Devices), and the time‐correlated single‐photon counting (TCSPC) data were gathered with a HydraHarp 400 controller from PicoQuant. The system exhibited an impressive overall time resolution of better than 200 ps. The histograms generated were analyzed using the SymphoTime64 software from PicoQuant, employing the Levenberg‐Marquardt iteration algorithm. A comprehensive discussion of the results can be found in Section 2.1.

### In Situ Light Illumination and X‐Ray Photoelectron Spectroscopy (XPS)

To confirm the quality of these crystals, in situ light illumination and XPS characterization of FA‐rich and MA‐rich samples were conducted to determine the relative atomic ratio. The XPS measurements were carried out using a multi‐probe ScientaOmicron ultrahigh vacuum chamber operated at 10^−9^ mbar. The system is equipped with a XM1000 monochromatic Al‐Kα (1486.6 eV) X‐ray source (15 kV, 26 mA) and a Sphera II EAC 125 Argus hemispherical analyzer with a 7‐channeltron detector positioned normal to the sample without charge neutralization. Photoelectrons were collected at an angle of 80° relative to the sample analyzer line. The spectra were recorded at a constant pass energy which was 50 eV for survey scans and 20 eV for high resolution scans. The binding energy of the target elements was calibrated to the C 1s peak at 284.6 eV. Atomic ratios of the detected elements at the surface of the samples were determined from the intensity of their main photoemission peaks: Pb 4f, I 3d, O 1s, N 1s, and C 1s. Samples were transferred directly to UHV with a minimal time of ambient exposure and measured without further preparation. CasaXPS software was used for all peak fitting and analysis with mixed Gaussian/Lorenztian peaks (30/60) and Shirley‐type background function.

For the illumination part of the experiment, a solar simulator (350–1100 nm, HAL‐320, Asahi Spectra) with compact 300 W xenon light source, located outside of the XPS chamber, was used to produce simulated sunlight AM 1.5G irradiation (100 mW cm^−2^) to in situ illuminate the samples through a transparent quartz window. After each cycle of light illumination, the samples were transferred for XPS measurement without a vacuum break.

### For Nitrogen (N_2_) Environment XPS Results

XPS was carried out on the same samples before and after light exposure of white light in a solar simulator (derya lab stability testing station) of 8 h in a sealed N_2_ vial, and under air mass (AM) of 1.5, 1800 s of X‐ray exposure.

### 4D Ultrafast Scanning Electron Microscopy (4D‐USEM)

A femtosecond pulsed fiber laser (Clark‐MXR) with an infrared (IR) wavelength of 1030 nm was coupled with a modified scanning electron microscope (SEM, QUANTA 650). The IR beam was guided to a beam splitter to divide the beam into two beams that are directed to nonlinear crystals for second and third‐harmonic generators (SHG, THG) to yield beams with wavelengths of 515 nm (green) and 343 nm (UV), respectively. The 515 nm beam was directed to the inside of the SEM chamber to pump the sample, while the UV beam was directed toward the gun source in the microscope to generate pulsed electrons, rather than the thermal continuous electron in conventional SEM, for probing the sample. Then, SEs were collected through a detector to form an image. The time difference between the pump and probe beam was controlled mechanically through an optical delay stage to add an extra travelling path to the pump beam. The filament current of the SEM was set to 0 A to suppress thermionic emission, with accelerating voltage of 30 kV for electron beam. The images were acquired with integration of 64 frames and 300 ns dwell time at each pixel to enhance signal‐to‐noise ratio. All experiments were conducted at a repetition rate of 8.33 MHz to affirm full relaxation of the specimen before the excitation of the next optical‐pulse, providing a time window of 125 ns between two pulses. Similar pump power was used for both composition ranging from (0.40–0.18 mW) depending in the sample quality to avoid damage of the pump.

### Density Functional Theory (DFT) Calculations

Density functional theory (DFT) calculations were carried out using the projector‐augmented wave (PAW) method as implemented in the VASP code.^[^ ^51^, ^52^ ^]^ The generalized gradient approximation (GGA) with the Perdew‐Burke‐Ernzerhof (PBE) exchange‐correlation functional was used. A uniform 6 × 6 × 6 *k*‐mesh grid in the Brillouin zone was employed to optimize the crystal structure of bulk tetragonal‐phase MAPbI_3_ cubic‐phase FAPbI_3_ and mixed cation (where the different cations were located diagonally), and a 2 × 2 × 1 *k*‐mesh was used for the MAPbI_3_, FAPbI_3_ and mixed cations slabs. The MAPbI_3_, FAPbI_3,_ and mixed‐cations slab model had a (2 × 2) lateral periodicity with exposed (001) surface and the slab replica was separated by ≈20 Å of vacuum. The plane‐wave basis set cutoffs of the wave functions were set at 500 eV for the bulks and 450 eV for slabs. The atomic positions of all structures were fully relaxed until the supercells had forces on each atom less than 0.01 eV Å^‐1^. The energy barriers for I^−^ and FA^+^ (or MA^+^) ion migration were calculated by the differences between the total energy of the ground state of MAPbI_3_, FAPbI_3_ and mixed cations and those at the saddle point that appeared in the diffusion pathway. The I**^−^** and FA^+^ (or MA^+^) ion migrations were investigated using the nudged elastic band (NEB) and constrained energy minimization methods.

## Conflict of Interest

The authors declare no conflict of interest.

## Supporting information

Supporting Information

## Data Availability

The data that support the findings of this study are available from the corresponding author upon reasonable request.
