# Supplementary material for: Mapping Surface‐Defect and Ions Migration in Mixed‐Cation Perovskite Crystals
Source: Adv Sci (Weinh). 2024 Aug 29;11(40):2404468. doi: 10.1002/advs.202404468 (PMC11516060; doi:10.1002/advs.202404468)
Supplement: Supplementary file 1 — Supporting Information [file ADVS-11-2404468-s001.docx]

**Supplementary Information**

**Mapping Surface-Defect and Ions Migration in Mixed-Cation Perovskite Crystals**

Razan O. Nughays^1^, Khulud Almasabi^2,3^, Sarvarkhodzha Nematulloev^1^, Lijie Wang^1^, Tieyuan Bian^4^, Issatay Nadinov^1^, Bahaaeddin Irziqat^5^, George T Harrison^1,5^, Shadi Fatayer^5^, Jun Yin^4^, Osman M. Bakr^2, 3^ & Omar F. Mohammed^*1,2^

^1^Advanced Membranes and Porous Materials Center (AMPM), Division of Physical Science and Engineering, King Abdullah University of Science and Technology, Thuwal 23955-6900, Kingdom of Saudi Arabia.

^2^KAUST Catalysis Center, Division of Physical Sciences and Engineering, King Abdullah University of Science and Technology, Thuwal 23955-6900, Kingdom of Saudi Arabia.

^3^Functional Nanomaterials Lab, Division of Physical Sciences and Engineering, King Abdullah University of Science and Technology, Thuwal 23955-6900, Kingdom of Saudi Arabia.

^4^Department of Applied Physics, The Hong Kong Polytechnic University, Kowloon 999077 Hong Kong, P. R. China.

^5^KAUST Solar Center (KSC), Division of Physical Science and Engineering, King Abdullah University of Science and Technology, Thuwal 23955-6900, Kingdom of Saudi Arabia.

Email: [*omar.abdelsaboor@kaust.edu.sa*](mailto:omar.abdelsaboor@kaust.edu.sa)

Keywords: Electron-imaging, surface, ion migrations, perovskites, DFT

1. **Surface inspection using static SEM, DFT and TRPL for defects formation:**


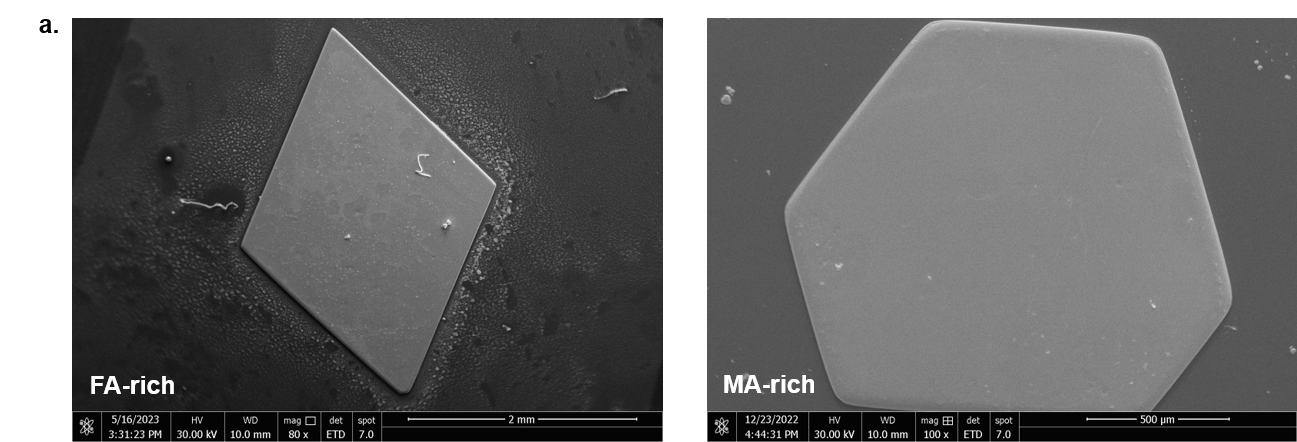


**Figure S1**

Static SEM images for a. FA-rich in and b. MA-rich showing the shape and surface quality of the two compositions of mixed-cation single crystals. The images are obtained using service mode with a filament current of 0 A, 300 pA pulsed electrons, 300 dwell time and 64 number of frames.


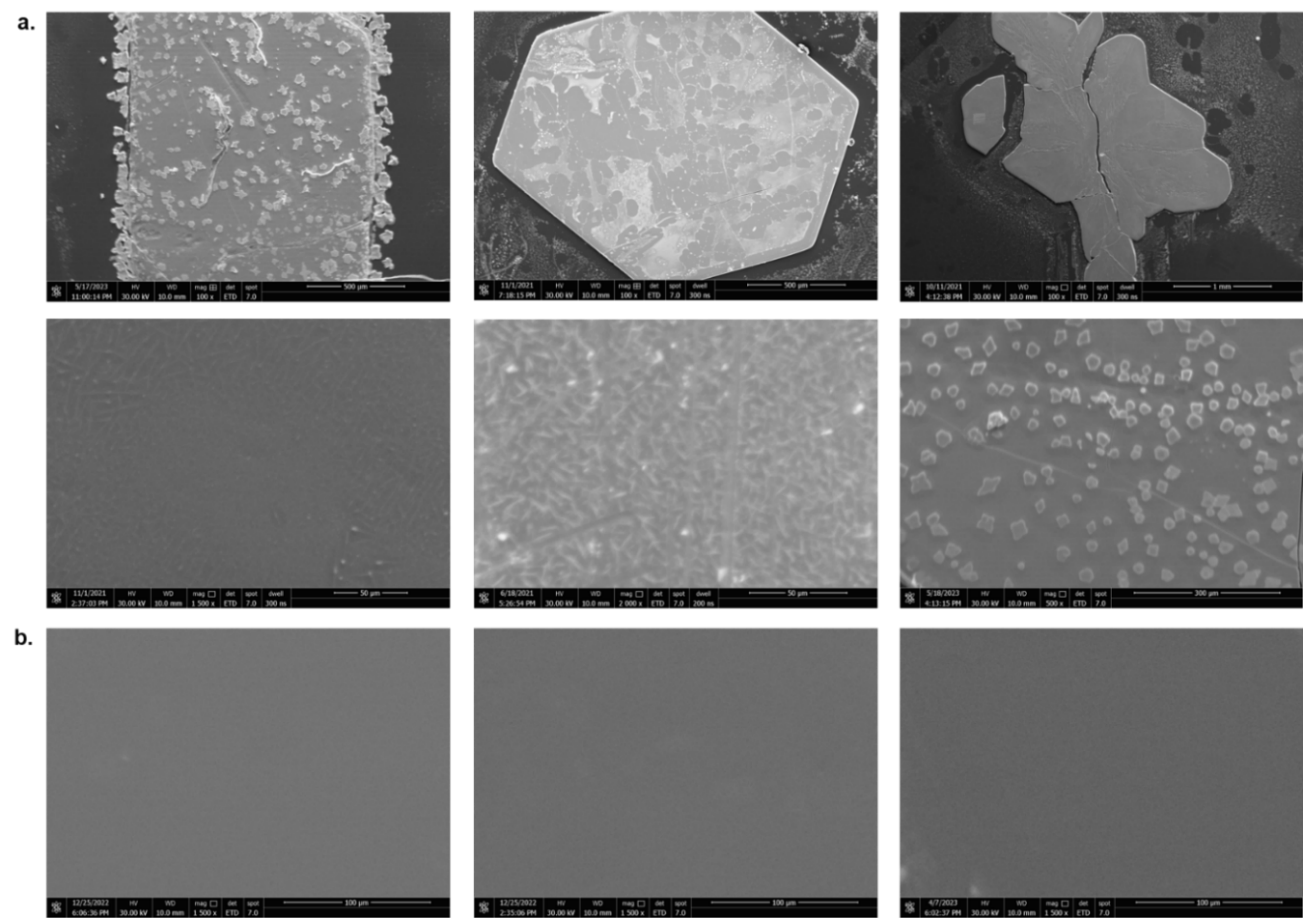


**Figure S2**

a) Images obtained by static mode in SEM shows poor surface quality and shape for the crystals. Solvent residue, particles and degradation are shown in these images. These areas have been avoided for image acquisitions. b) Clean, uniform and flat area selected for 4D-USEM measurements.

**Table S1.** Calculated defect formation energies for V_FA_ in FAPbI_3_ and V_MA_ in MAPbI_3_ under I-rich, moderate, and Pb-rich conditions. The calculations were performed at the GGA/PBE+vdW level of theory.

| **Defect formation energy (eV)** | **I-rich** | **Moderate** | **Pb-rich** |
| --- | --- | --- | --- |
| V_FA_ | 0.72 | 1.18 | 1.66 |
| V_MA_ | 0.29 | 0.68 | 1.25 |


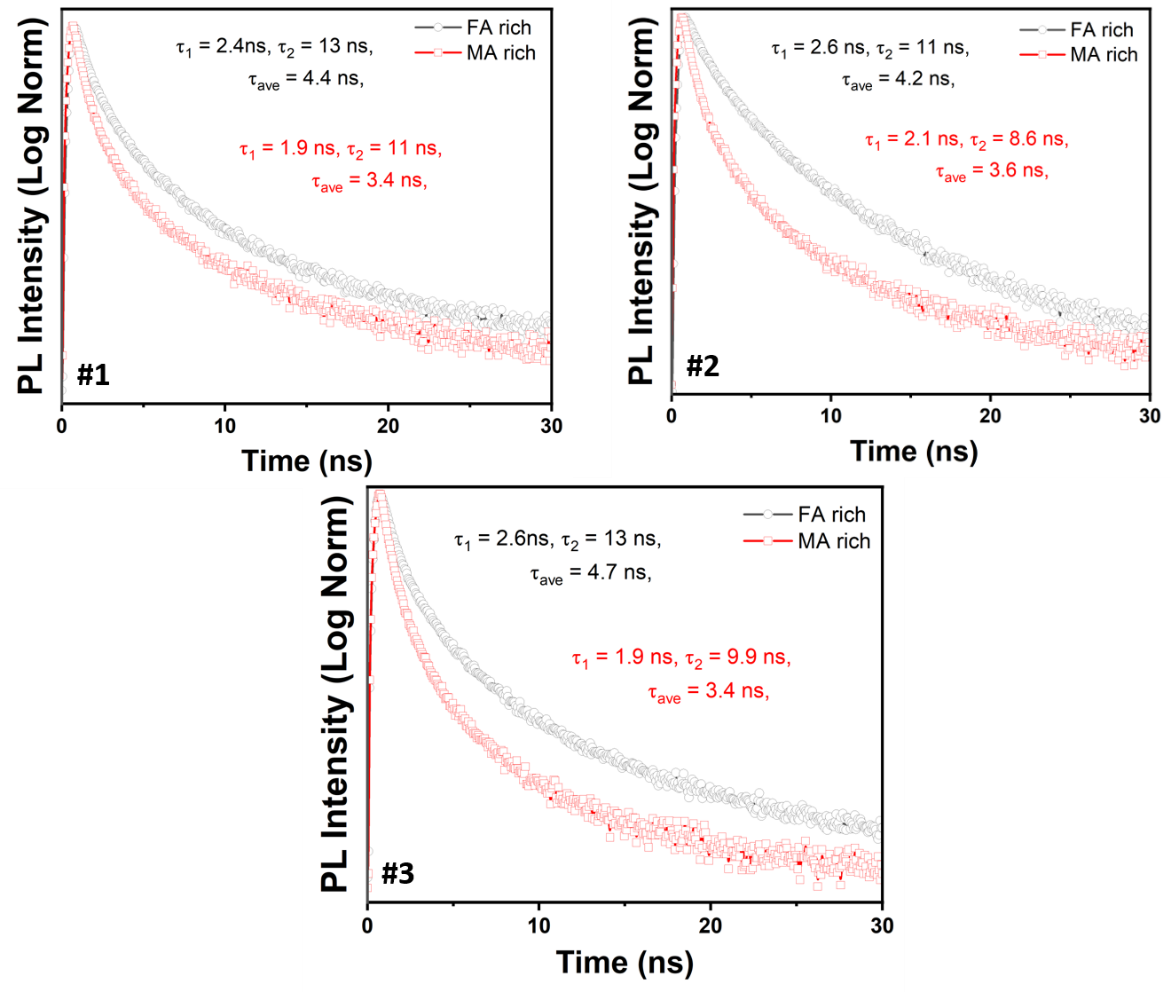


**Figure S3**

TRPL on three different spots on the samples showing relatively faster decay for MA-rich samples.

1. **Additional 4D-USEM measurements:**


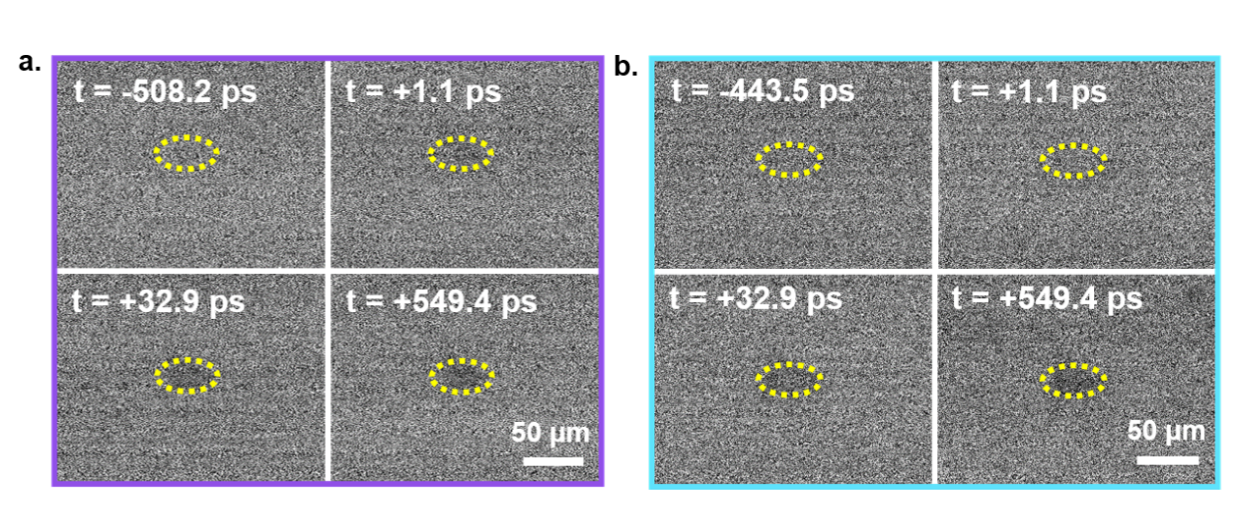


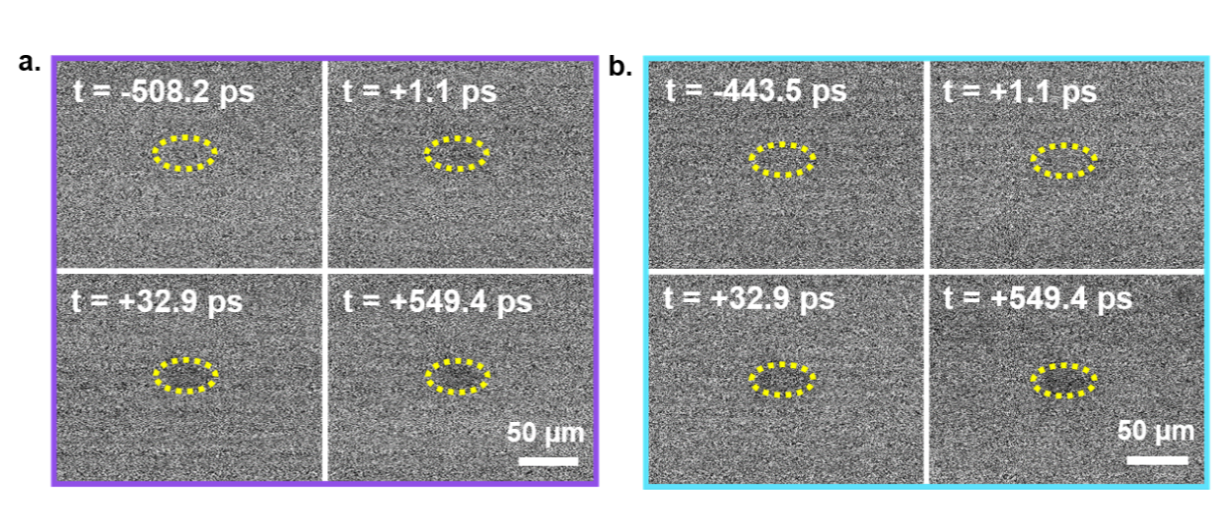


**Figure S4**

Time-resolved images for a) FAPbI_3_ and b) MAPbI_3_ at different time delay. Dark signals formed after photo-excitation at +1.1 ps.


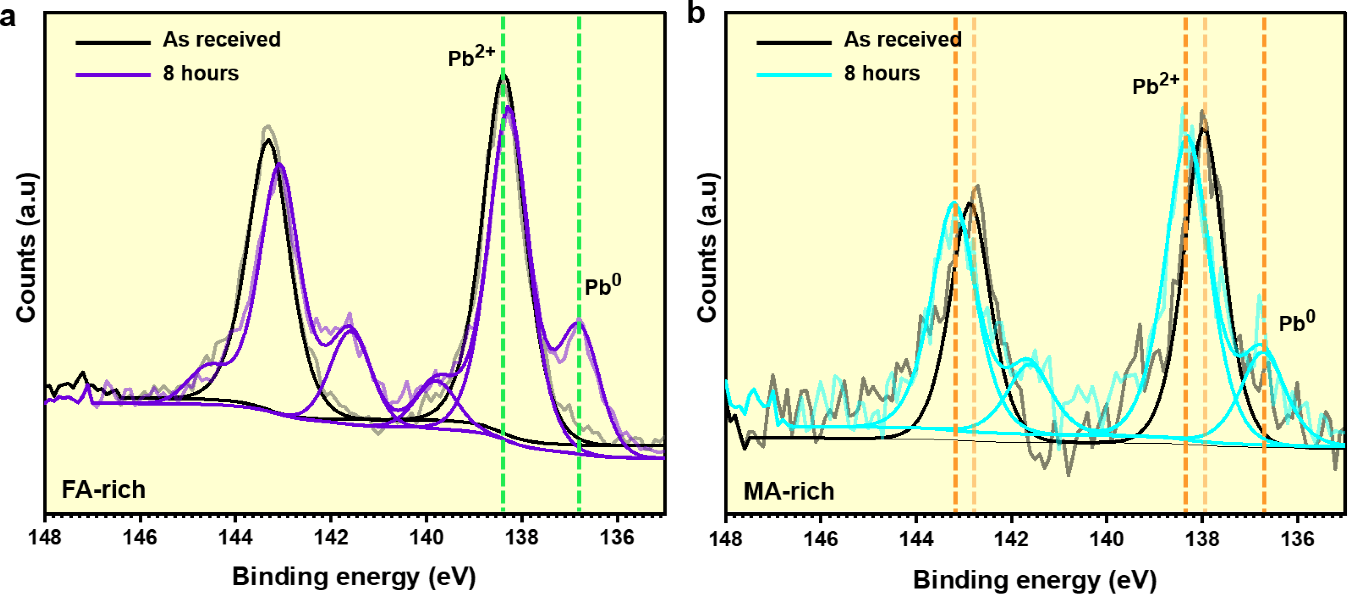


**Figure S5**

X-ray photoelectron spectroscopy of FA-rich (a) and MA-rich (b) on as-received sample (in black) and after light exposure (on purple and cyan, respectively). We can see a new peak formed at ~136.7 for Pb­^0^ after light exposure in the two samples.

| Sample | Iodide %At. Conc. (As-received ) | Iodide %At. Conc. (After Light) | Pb/I Ratio  (As-received) | Pb/I Ratio (After Light) |
| --- | --- | --- | --- | --- |
| FA-rich | 34.79 | 34.54 | 0.380 | 0.398 |
| MA-rich | 33.06 | 31.57 | 0.359 | 0.388 |

**Table S2**. XPS analysis for iodide before and after 8-hourse of light exposure


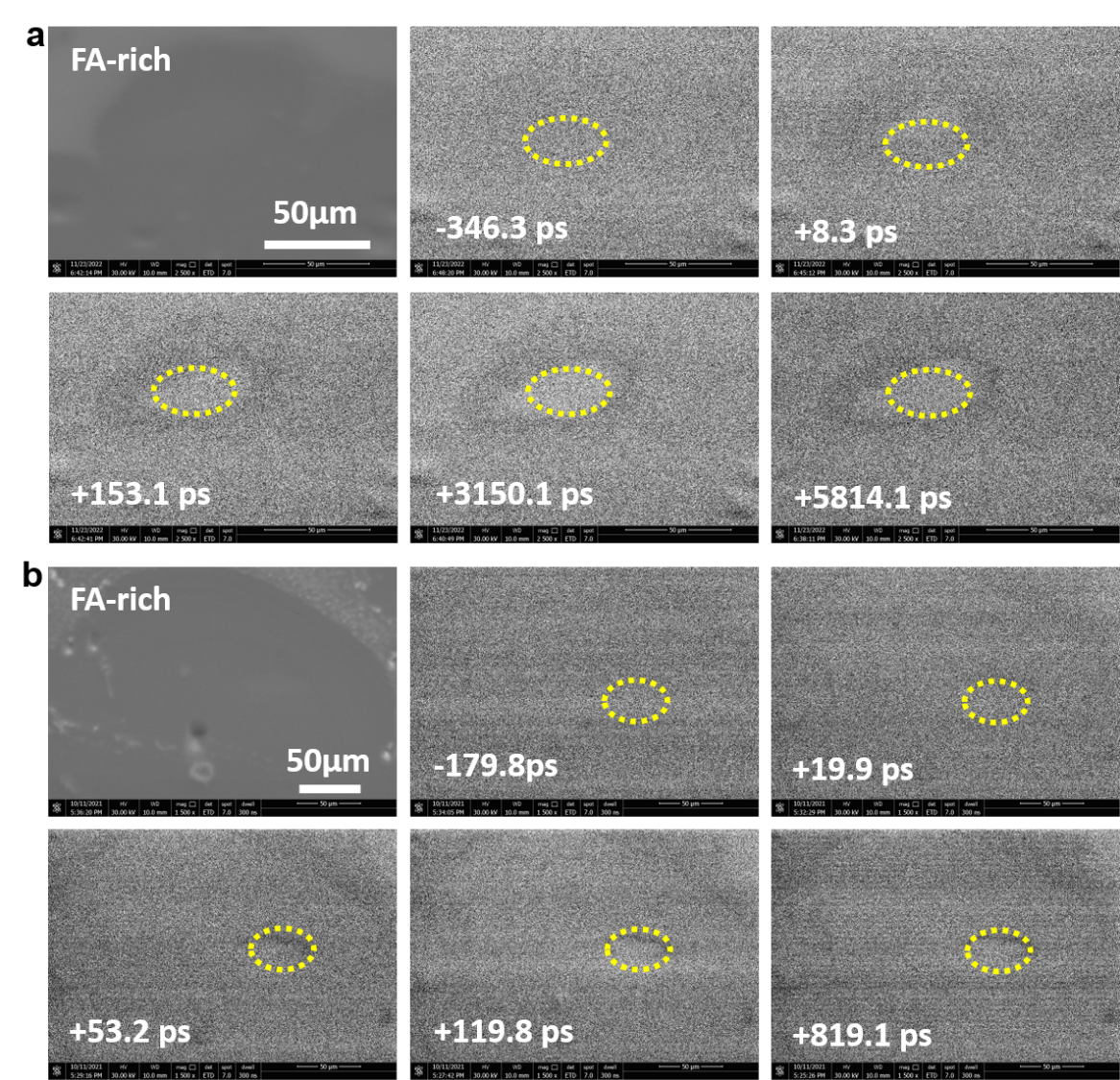


**Figure S6**

Time-resolved SE images for FA-rich at two different areas of poor samples. The first images in **a and b** show the surface quality where the experiment have been performed. Bright contrast have been formed. The signal contrast in this case is an artificial due the poor surface quality. The yellow ellipse represent the laser footprint of the excitation beam.


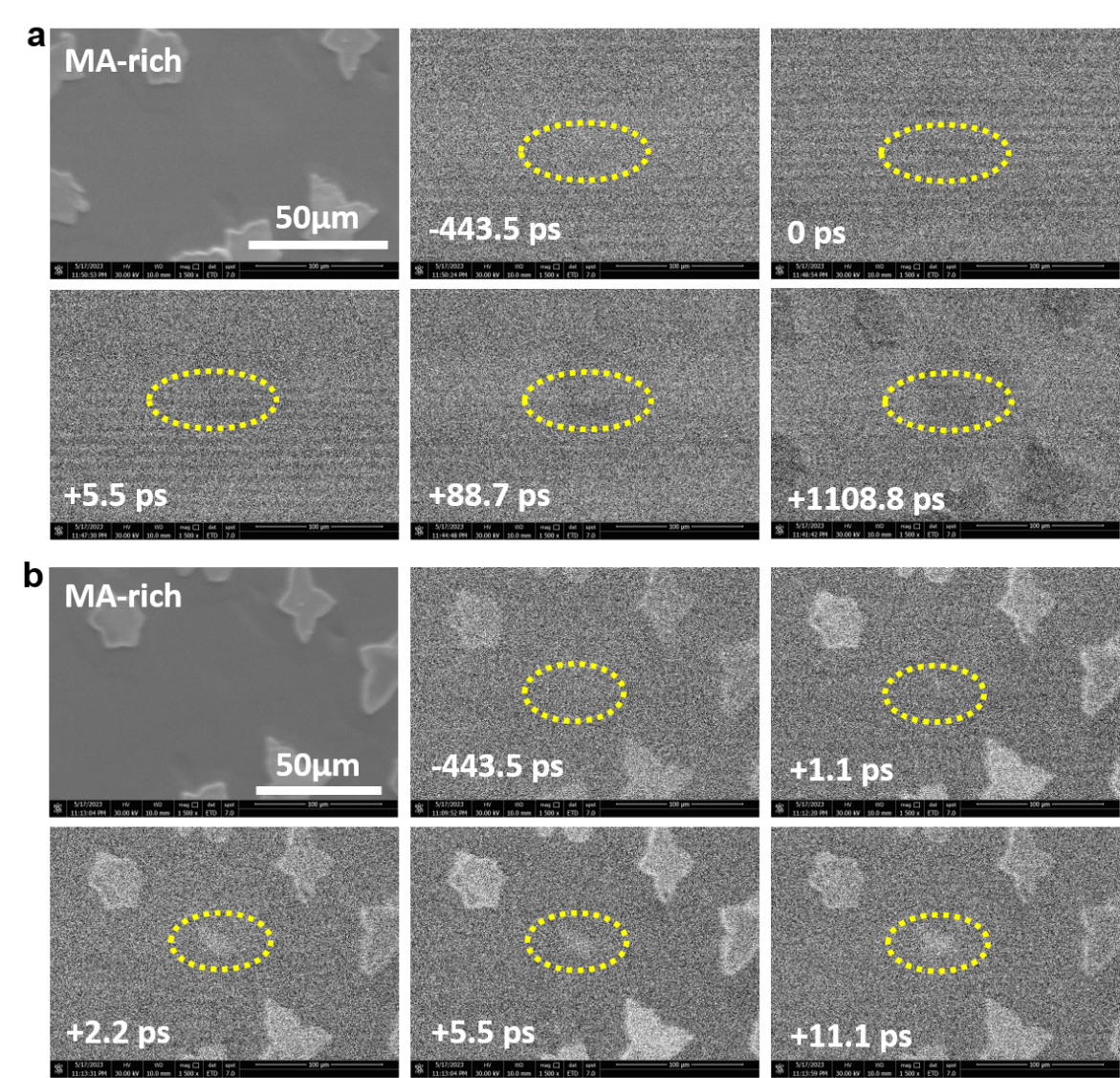


**Figure S7**

4D-USEM images for MA-rich on two areas with bad surface-quality as can be seen in **a and b**. Dark contrast (in the top) and bright contrast (in the bottom) have been formed. The signal formation was too slow and weak because of the poor quality.


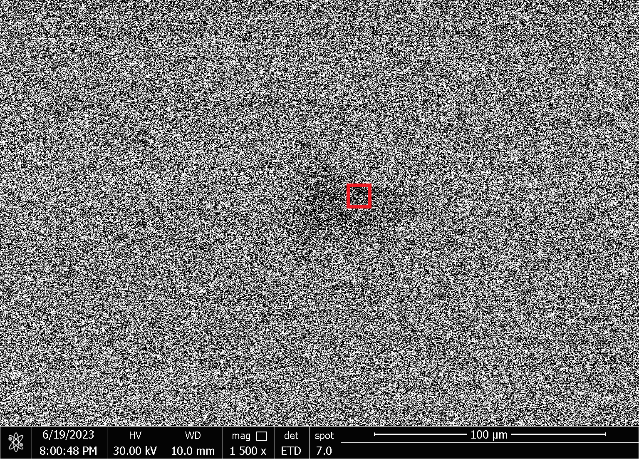


**Figure S8**

4D-USEM image with a dark contrast, the red square show the area where the dynamics were extracted.

1. **DFT calculations:**


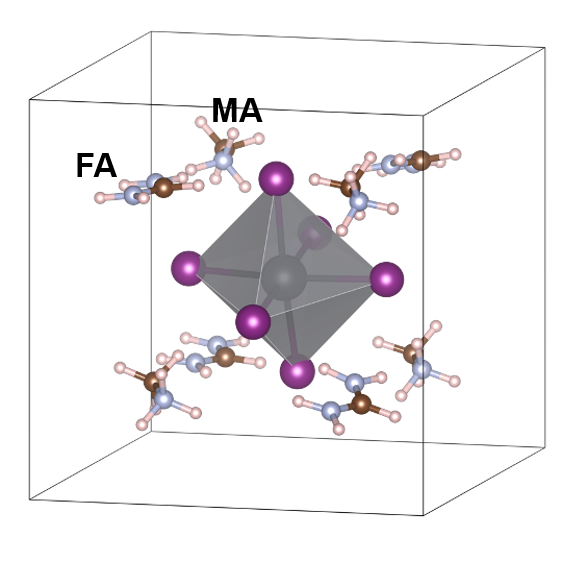


**Figure S9**

Unit cell of the mixed cation system, FA_50_MA_50_PbI_3_, which have been used for the DFT calculations.


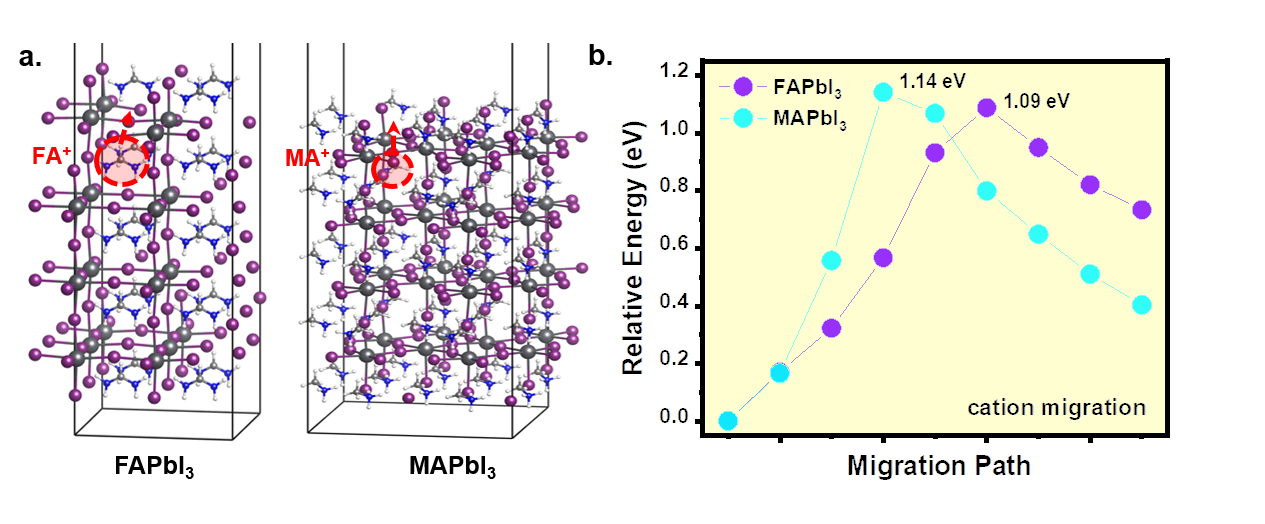


**Figure S10**

a) Side-view of migration path for the cations ion (FA^+^ and MA^+^) from subsurface to top surface in the pritstine-FAPbI_3_ and pristine-MAPbI_3_ for another slap. The red arrow indicates. b) Energy barriers for FA^+^ and MA^+^, where both cations exhibited a similar migration behavior and close value of migration barrier. The DFT calculations were performed at the GGA/PBE+vdW level of theory.


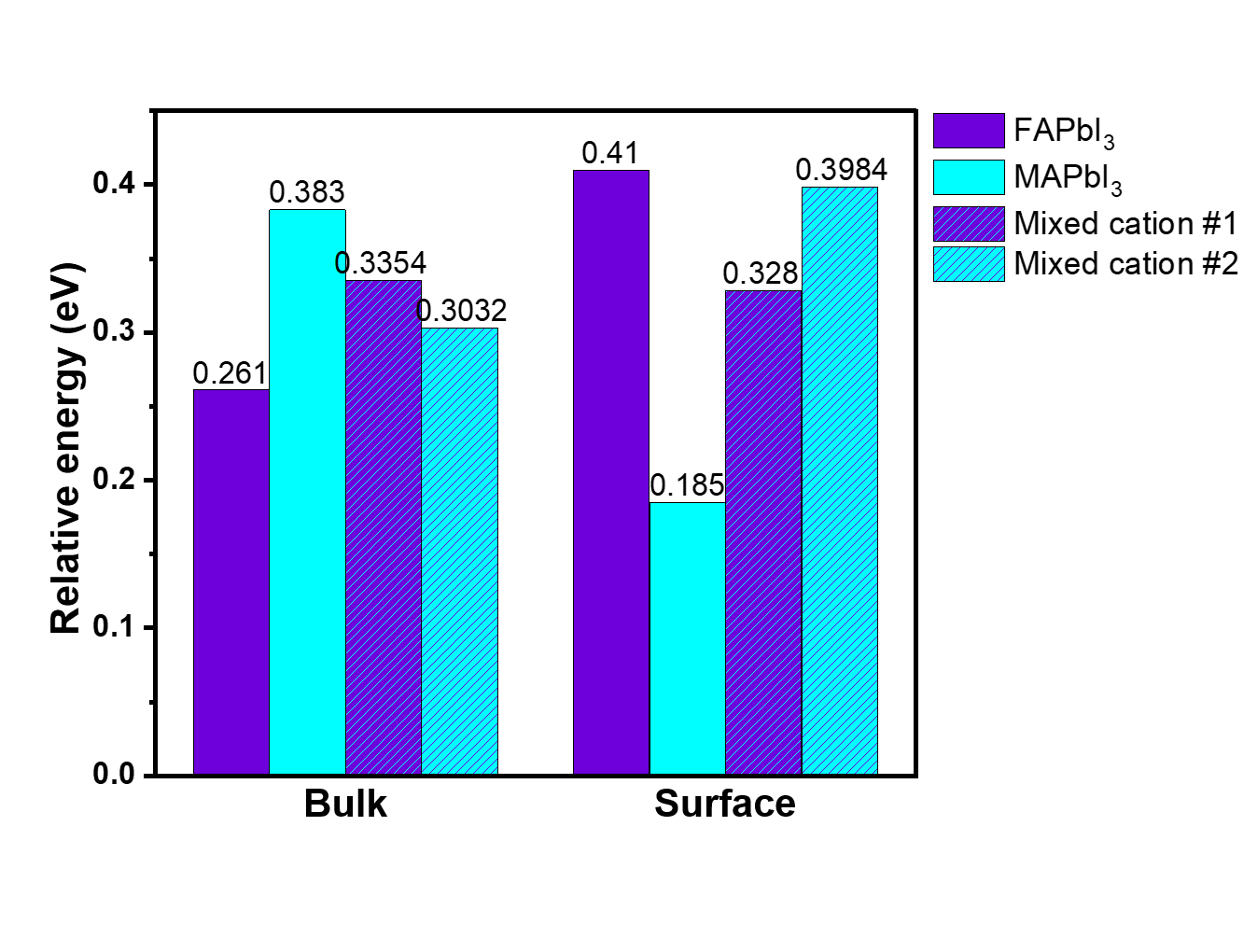


**Figure S11**

The highest and lowest energy barrier for iodine ions in bulk and surface for FAPbI_3_ (purple), MAPbI_3_ (cyan) and the mixed-cation with its two paths (diagonal lines).


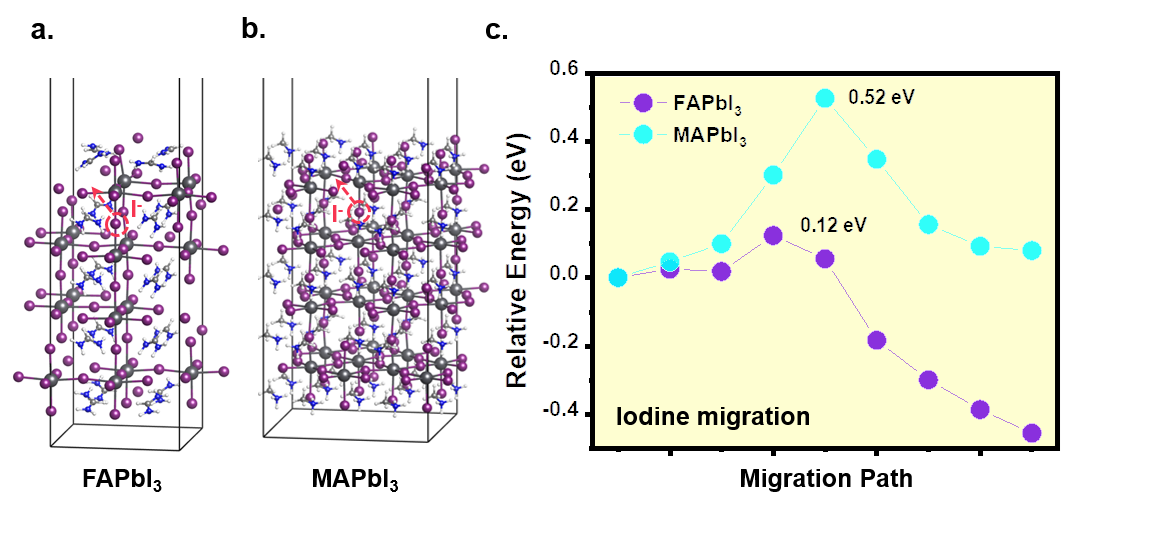


**Figure S12**

Another side-view of migration path for I**^-^** ion from bulk to top surface in a) FAPbI_3_ and b) MAPbI_3_. The red arrow indicates I**^-^** ion diffusion pathways. c) Diffusion energy barriers I**^-^** ion in FAPbI_3_ and MAPbI_3_. The calculations were performed at the GGA/PBE+vdW level of theory.
